# Practice variation in the informed consent procedure for thrombolysis in acute ischemic stroke: a survey among neurologists and neurology residents

**DOI:** 10.1186/s12910-021-00684-6

**Published:** 2021-08-25

**Authors:** Valentijn J. Zonjee, Jos P. L. Slenders, Frank de Beer, Marieke C. Visser, Bastiaan C. ter Meulen, Renske M. Van den Berg-Vos, Sander M. van Schaik

**Affiliations:** 1 Department of Neurology, OLVG, Jan Tooropstraat 164, 1061 AE Amsterdam, The Netherlands; 2 Department of Neurology, Spaarne Gasthuis, Haarlem, The Netherlands; 3 Department of Neurology, Amsterdam UMC, Location VUmc, Amsterdam, The Netherlands; 4 Department of Neurology, Amsterdam UMC, Location AMC, Amsterdam, The Netherlands

**Keywords:** Clinical practice, Acute ischemic stroke, Thrombolysis, tPA, Informed consent, Acute stroke therapy

## Abstract

**Background:**

Obtaining informed consent for intravenous thrombolysis in acute ischemic stroke can be challenging, and little is known about if and how the informed consent procedure is performed by neurologists in clinical practice. This study examines the procedure of informed consent for intravenous thrombolysis in acute ischemic stroke in high-volume stroke centers in the Netherlands.

**Methods:**

In four high volume stroke centers, neurology residents and attending neurologists received an online questionnaire concerning informed consent for thrombolysis with tissue-type plasminogen activator (tPA). The respondents were asked to report their usual informed consent practice for tPA treatment and their considerations on whether informed consent should be obtained.

**Results:**

From the 203 invited clinicians, 50% (n = 101) completed the questionnaire. One-third of the neurology residents (n = 21) and 21% of the neurologists (n = 8) reported that they always obtain informed consent for tPA treatment. If a patient is not capable of providing informed consent, 30% of the residents (n = 19) reported that they start tPA treatment without informed consent. In these circumstances, 53% of the neurologists (n = 20) reported that the resident under their supervision would start tPA treatment without informed consent. Most neurologists (n = 21; 55%) and neurology residents (n = 45; 72%) obtained informed consent within one minute. None of the respondents used more than five minutes for informed consent. Important themes regarding obtaining informed consent for treatment were patients’ capacity, and medical, ethical and legal considerations.

**Conclusion:**

The current practice of informed consent for thrombolysis in acute ischemic stroke varies among neurologists and neurology residents. If informed consent is obtained, most clinicians stated to obtain informed consent within one minute. In the future, a shortened information provision process may be applied, making a shift from informed consent to informed refusal, while still considering the patient’s capacity, stroke severity, and possible treatment delays.

**Supplementary Information:**

The online version contains supplementary material available at 10.1186/s12910-021-00684-6.

## Background

An increasing number of patients diagnosed with acute ischemic stroke are treated with intravenous thrombolysis (IVT) using tissue-type plasminogen activator (tPA) [1, 2]. In acute ischemic stroke, ‘time is brain’: each minute an acute ischemic stroke patient with large vessel occlusion is not treated, approximately 2 million neurons are lost [3, 4]. However, 1–6% of tPA treatments are complicated by a symptomatic intracerebral hemorrhage (ICH) [5, 6]. In 2002, the European Medicine Agency licensed tPA treatment for acute ischemic stroke within only 3 h after stroke symptom onset [7], but current American and European stroke guidelines recommend that treatment must be started within 4.5 h of ischemic stroke onset, in absence of contraindications [8, 9]. Only in specific cases of acute ischemic stroke with favorable stroke imaging characteristics, tPA treatment can be started within 9 h after onset [9, 10].

The procedure of informed consent for tPA treatment in acute ischemic stroke faces various challenges: 1) tPA treatment’s efficacy increases with earlier administration [11], 2) the decision-making process takes place in an acute setting, 3) in most cases, an alternative therapy is lacking [12], and 4) obtaining consent can be difficult in stroke patients with neurological deficits (e.g. aphasia) [13]. However, patient autonomy is considered a ground principle in modern medicine and informed consent was constituted to protect this principle.

A valid informed consent procedure requires adequate information provision, absence of coercion and adequate decision-making capacity [14]. The requirements of this procedure are dependent on how much time is available, the severity and risks of complications, if alternative treatments are available and what the professional consensus is concerning this treatment [15]. For most study interventions and elective surgeries, written informed consent is obtained [16], whereas for standard of care or emergency treatment, informed consent is usually obtained verbally or might even be waived [17]. Effectively informing patients with acute ischemic stroke about treatment risks and benefits can be challenging, for which a standardized informed consent procedure could be useful [18–20].

In the Netherlands, the law prescribes that “the consent of the patient is required for actions to be performed in the implementation (performance) of the medical treatment agreement” [21]. Consent can be provided verbally, but “upon the request of the patient, the care provider shall in any event put down in writing the consent given by the patient for medical actions of a far-reaching nature” [22]. Consent can be obtained from legal representation if “a patient who has reached the age of majority and who cannot be regarded as being capable of making a reasonable appreciation of his interests in the matter” [23]. In case of an emergency, the Dutch Civil Law states that actions “may be performed without the consent of this person if there is no time to request his consent because immediate performance of the action is clearly necessary to prevent serious harm to the patient” [24].

It is not fully known if and how informed consent for thrombolysis is obtained in clinical practice, as only a few studies investigated informed consent practice for thrombolysis in acute ischemic stroke. A previous study examined protocols of informed consent practice for tPA, and found that the informed consent procedure differed per hospital [25]. More recent literature suggested that there is no consensus on if informed consent should be obtained for tPA treatment in acute ischemic stroke. About half of the respondents in that study, which were clinicians involved in acute stroke care, believed informed consent is never required, whereas the other half believed some form of informed consent is necessary [26, 27]. Since these studies pooled the practice and considerations of both neurologists and emergency physicians, the clinical practice of informed consent for thrombolysis among clinicians practicing neurology remains unknown.

The aim of this study was to examine the current informed consent procedure for thrombolysis among neurology residents and neurologists involved in acute stroke care in high-volume stroke centers in the Netherlands.

## Methods

### Design

Questionnaires concerning the informed consent practice for thrombolysis were distributed among neurology residents and attending neurologists involved in acute stroke care in four high volume stroke centers in the Netherlands. Two academic hospitals and two non-academic hospitals were examined: Amsterdam University Medical Centers (UMC) location Academic Medical Center (AMC) and location VU Medical Center (VUmc), OLVG Amsterdam, and Spaarne Gasthuis Haarlem. Clinicians were approached using a modified Dillman recruitment method [28]. The questionnaire was accessible from 8 July 2019 until 22 September 2019. If necessary, clinicians were sent a reminder two weeks after the initial invitation. This study was reviewed and approved by the OLVG’s institutional board.

### Questionnaires

Questionnaires regarding the consent practice for *on-label* use of tPA in acute ischemic stroke were developed, and were checked for face validity by an ethicist and two vascular neurologists. Respondents were asked to report their practice for patients who were eligible for treatment within 4.5 h and had no contra-indications for treatment. The administration of tPA in the included hospitals was generally performed by neurology residents and not by neurologists. Therefore, two separate questionnaires were developed: one intended for neurology residents, the other intended for attending neurologists. Both questionnaires focused on the general consent practice of the resident or on the resident under the neurologist’s supervision.

In order to investigate the prerequisites of informed consent, the questionnaires addressed whether explicit consent is obtained, how much time is spent on informed consent, the general content of information provision and the patients’ decision making capacity. Furthermore, informed consent by proxy and experienced treatment delays because of informed consent were addressed. In open-ended questions, clinicians were asked to note their considerations on when to obtain consent. Respondents were asked to report their usual informed consent practice.

For most questions, a 4-point Likert scale was used ranging from “never” to “always”. Attending neurologists were able to select the option “unknown”, if the practice of the resident under their supervision was unknown.

The questionnaires were distributed and completed using Castor Electronic Data Capture (EDC) [29]. The complete questionnaires can be found in the Additional file 1. In the questionnaire invitation, clinicians were informed about the study and that their responses were anonymous. Informed consent was considered to be provided upon questionnaire completion. This study was reviewed and approved by the local institutional review board and was conducted in accordance with the Declaration of Helsinki [30].

### Analysis

Questionnaires for neurologists and residents were analyzed separately. Descriptive statistics were used to describe the frequencies and distribution of answers among the different categories. Data were tested for normality by means of visual inspection combined with the Shapiro–Wilk test of normality. The chi-square test was used to investigate differences in response categories of the reported informed consent practice between academic and non-academic respondents. Responses on whether explicit informed consent is obtained were dichotomized into always obtaining informed consent and not always obtaining informed consent: response options “never”, “sometimes” and “often” were transformed to ‘do not always obtain consent’; the response “always” was transformed to ‘always obtain consent’. Univariable logistic regression analyses were performed to determine independent factors that contributed to always obtaining informed consent. The following a priori predictors of interest were identified: experience and age of the clinician, and academic or non-academic setting. A two-tailed p value of p < 0.05 was considered statistically significant. Statistical Package for the Social Sciences (SPSS) version 22 was used for all quantitative analyses.

Free-text data from open ended questions were coded and categorized for thematic analysis and were used to analyze clinicians’ comments on the consent procedure. Codes were identified, indexed and transcending themes were identified using MAX Qualitative Data Analysis (QDA) version 2007.

## Results

### Characteristics

A total of 203 clinicians were invited to participate in this study, of whom 101 (50%) completed the questionnaire. All participants had experience with tPA administration or supervision of tPA administration (Table 1). The neurology residents’ age was 31.2 years (SD 3.1) and reported 5.0 years of clinical experience (SD 2.9). The age of the neurologists was 44.5 years (IQR 10) with 17 years of clinical experience (IQR 12.3).

**Table 1:** Respondent characteristics in academic and non-academic hospitals

Respondent characteristics	Total (n = 101)	Academic (n = 46)	Non-academic (n = 55)
Residents in neurology, n (%)	63 (62)	34 (74)	29 (53)
Attending neurologists, n (%)	38 (38)	12 (26)*	26 (47)
Male, n (%)	50 (50)	22 (48)	28 (51)
Age (in years), median (IQR)	33 (11)	36 (16)	33 (6)
Experience (in years), median (IQR)	7 (8.5)	6.5 (22)	8 (13)
tPA administered in:^‡^	Total (n = 63)	Academic (n = 34)	Non-academic (n = 29)
1–25 patients, n (%)	18 (29)	10 (29)	8 (28)
> 25 patients, n (%)	45 (71)	24 (71)	21 (72)
tPA supervised in:^†^	Total (n = 38)	Academic (n = 12)	Non-academic (n = 26)
1–25 patients, n (%)	7 (18)	5 (42)	2 (7.7)
> 25 patients, n (%)	31 (82)	7 (58)	24 (92)*
tPA refused by:	Total (n = 101)	Academic (n = 46)	Non-academic (n = 55)
0 patients, n (%)	44 (44)	25 (54)	19 (35)
1–5 patients, n (%)	51 (51)	19 (41)	32 (58)
> 5 patients, n (%)	1 (1)	0 (0)	1 (2)
Unknown, n (%)	5 (5)	2 (4)	3 (6)

*tPA* tissue-type Plasminogen Activator

^*^p < 0.05, ^‡^Neurology residents were asked to report the number of patients that they treated with tPA, ^†^attending neurologists were asked how many tPA treatments they had supervised

### Content of information provision

The majority of the neurology residents reported that they always inform their patient about the diagnosis ischemic stroke (n = 55; 87%), treatment mechanism of action (n = 49; 78%), and about the benefits (n = 38; 60%) and risks (n = 42; 67%) of tPA administration, before starting tPA treatment. If residents inform stroke patients about the risks of tPA, all residents reported to mention the risk for ICH as a complication of treatment.

Most of the neurologists (n = 29; 76%) reported that, under their supervision, the patient is always provided with information about their diagnosis. About half of the neurologist reported that the resident always discusses the risks and benefits of treatment (n = 20; 53% and n = 21; 55% respectively). About half of the neurologists (n = 21, 55%) reported that treatment mechanism of action is always discussed with the patient. Eighty-two percent of the neurologists (n = 31) expected that residents provide the same information to every patient eligible for tPA treatment.

Analysis of response categories using the chi-square test demonstrated no significant differences in information provision between academic and non-academic practicing residents and neurologists.

### Informed consent practice

Residents—A third of the neurology residents (n = 21) reported that they always obtain explicit consent before starting tPA treatment (Table 2). Six percent (n = 4) reported that they never obtain explicit informed consent for tPA treatment. Univariable logistic regression analysis demonstrated that a year increase in age of the residents resulted in being 18% less likely to always obtain explicit informed consent (OR, 0.82; 95%CI, 0.68–0.99; p = 0.039). None of the residents reported that they spend more than 5 min on informed consent. Five percent (n = 3) reported to spend no time, 67% (n = 42) reported to spend 0–1 min, and 29% (n = 18) spend 1–5 min on providing information and obtaining consent. Eighty-three percent of the residents (n = 52) reported that obtaining informed consent sometimes, often or always causes a delay in starting treatment.

**Table 2:** Reported consent practice from residents in neurology and attending neurologists, grouped per question

Question	Response	Residents n = 63 (n, %)	Neurologists* n = 38 (n, %)
*Before starting tPA treatment, the patient is asked for explicit consent:*	Always	21 (33)	8 (21)
	Often	25 (40)	10 (26)
	Sometimes	13 (21)	7 (18)
	Never	4 (6.3)	7 (18)
	Unknown	NA	6 (16)
*How much time is spent on information provision and informed consent?* †	0 min	3 (4.8)	2 (5.3)
	0–1 min	42 (67)	21 (55)
	1–5 min	18 (29)	15 (40)
	> 5 min	0 (0.0)	0 (0.0)
*If an acute ischemic stroke patient is unable to provide consent for tPA treatment:*	tPA treatment is started	19 (30)	20 (53)
	Proxy consent is obtained, if present in the ER	40 (64)	17 (45)
	Proxy consent is obtained, even if not present in the ER	4 (6.3)	1 (2.6)
*Is a patient diagnosed with acute ischemic stroke able to make a well considered decision regarding treatment?*	Always	1 (1.6)	1 (2.6)
	Often	13 (21)	10 (26)
	Sometimes	48 (76)	25 (66)
	Never	1 (1.6)	2 (6.5)
*Does informed consent cause a delay in treatment?*	Always	4 (6.3)	3 (7.9)
	Often	5 (7.9)	1 (2.6)
	Sometimes	43 (68)	27 (71)
	Never	11 (18)	3 (7.9)
	Unknown	NA	4 (11)

*ER* Emergency Room, *tPA* tissue Plasminogen Activator, *NA* not applicable

^*^Questions for neurologists were preceded by: “Under my supervision, …”. †Neurologists were asked how much time they consider necessary for informed consent

If a patient is not capable of providing consent, 64% of all neurology residents (n = 40) reported that they obtain proxy consent if a proxy is present in the emergency room (ER). Thirty percent (n = 19) reported to start tPA treatment without any form of consent in that situation. Forty neurology residents (64%) reported that they never discuss informed consent for tPA treatment in acute ischemic stroke with their supervising attending neurologist.

Neurologists—As shown in Table 2, neurologists’ responses on whether explicit consent is obtained were dispersed among all answer categories. Fifty-five percent of the neurologists (n = 21) reported that it is necessary to spend a maximum of 1 min on obtaining informed consent. None of the neurologists considered spending more than 5 min on obtaining informed consent for tPA necessary. Most neurologists (n = 31; 82%) reported that obtaining informed consent at least sometimes causes a delay in the start of tPA treatment. If a stroke patient eligible for tPA treatment is unable to provide consent, 53% of neurologists (n = 20) reported that, under their supervision, tPA treatment is commenced regardless of the availability of a proxy decision-maker.

Only a small minority of the responding clinicians judged that a patient with acute stroke is never (n = 3, 2%) or always (n = 2, 3%) capable to make a measured decision regarding tPA therapy.

### Considerations regarding informed consent

In free-text fields, clinicians reported various different considerations on whether informed consent for tPA treatment should be obtained and for what reasons. Patient’s capacity, ethical, medical and legal considerations were identified as transcending themes. The most frequently reported reason for obtaining informed consent was to inform about the risk of severe complications of treatment. This was reported for patients with minimal neurological deficits specifically, where complications of treatment could cause more damage relative to the initial impairments. Others reported the obligation, legally and morally, to inform the patient and ask permission for administration of this invasive treatment. Some stated that informed consent is no real cause for treatment delay.

However, others stated that informed consent for tPA treatment is not required. The most frequently reported reason for not obtaining informed consent was that thrombolysis is a standard treatment with a proven efficacy. Clinicians reported that because the decision-making takes place in an acute setting, they believe that patients have an impaired decision-making capacity. In addition, clinicians reported that they consider patients with neurological deficits such as a lowered consciousness, aphasia, or impaired cognitive function to have an impaired-decision making capacity and do not obtain informed consent in these cases. Some clinicians reported that starting tPA treatment is in the patient’s best interest and thus does not require informed consent. Many clinicians reported to inform the patient about the risks and benefits of treatment, but do not ask explicit consent for treatment. Table 3 shows an overview of the clinicians’ most reported considerations on obtaining consent.

**Table 3:** Clinicians’ considerations on obtaining informed consent for tPA treatment in patients with acute ischemic stroke

	Quote
Reasons for obtaining informed consent*	
Obliged to (legally and morally)	*“Patient’s autonomy comes first, even if I emphasize the urgency of treatment, the patients have the right to decide if they accept (the risks of) treatment. Because of the urgency of the situation I sometimes doubt patients’ capacity to make a measured decision, but I do not think this is a valid reason to refrain from obtaining informed consent.”* Resident 37
Risk for (severe) complications	
Invasive treatment	
No real cause for delay	
Reasons for not obtaining informed consent*	
Standard (and proven) treatment	*“It [acute ischemic stroke] is an acute situation, where a proven effective treatment can be administered (thrombolysis). In these cases I think that as a doctor you have to act in the medical interest of the patient, which means that without contra-indications or clear other medical reasons, the best treatment [thrombolysis] must be administered in this situation”* Neurologist 19
Acute situation (‘Time is brain’)	
Impaired decision-making capacity	
Act in the best interest of the patient	

^*^Respondents were asked for their considerations on obtaining informed consent in patients with acute ischemic stroke that were eligible for tPA treatment within the 4.5 h time-window without any contraindications

## Discussion

This study demonstrates that it is highly variable whether residents in neurology or neurologists involved in acute stroke care obtain informed consent before starting tPA treatment in patients with acute ischemic stroke. Furthermore, the procedure and content of informed consent, if performed, differs among clinicians as well and is dependent on circumstances such as neurological deficits and presence of family in the ER. However, if informed consent is obtained, all clinicians stated to obtain informed consent within five minutes.

In a previous survey study among clinicians involved in acute stroke care, it was demonstrated that a small percentage (26%) of the respondents who practiced neurology never required informed consent for thrombolysis [26]. Likewise, only 11% of our respondents, who all practiced neurology, reported that they never obtain informed consent for tPA treatment and no uniformity in practice was observed. Furthermore, if informed consent is obtained, it differed how the informed consent procedure is performed. If patients are not able to provide informed consent themselves, approximately 40% of the respondents reported that they start treatment without consent, while the rest attempts to obtain informed consent through a proxy. By stating their considerations in free-text fields on whether informed consent should be obtained, the respondents in our study further elucidated this observed variation in practice: some of the clinicians felt the legal and moral obligation to obtain consent, whereas others believed there was a medical reason to start treatment as fast as possible without a possible delay of informed consent, thus acting in the best interest of the patient. Most of these ethical, legal and medical themes are important recurring arguments that are supported by earlier studies [27]. The conflicting considerations within these themes might perhaps be due to the fact that informed consent for thrombolysis is not frequently discussed among neurologists and between neurologists and residents. Two-thirds of the residents in our study reported that they never discuss informed consent for thrombolysis with their supervisor, which might explain the differences between neurologists and residents in informed consent practice. This lack of discussion is supported by earlier studies, where most residents reported that they never received feedback on their informed consent communication in acute ischemic stroke and that ethical topics in general are rarely discussed with their supervisor [31, 32].

Based on our results and those of previous studies, we think it is doubtful whether valid informed consent for tPA treatment can be obtained or even should be obtained in patients with acute ischemic stroke [33, 34]. While most clinicians reported that they often or always inform the patient about stroke diagnosis, tPA mechanism of action, benefits and potential risks of tPA treatment, most of the clinicians stated to provide all this information within only one minute. Especially in a vulnerable patient that is suffering a stroke, it is unlikely that this is enough time to adequately communicate risks and benefits and obtain valid informed consent. A study that investigated the informed consent procedure in acute ischemic stroke found that nearly 3 min of discussing informed consent was required to create acceptable understanding of the risks and benefits of tPA treatment [20]. In that study, however, acceptable understanding of tPA treatment was judged on both patient’s and surrogate’s (family member or legal representative) understanding of the risks and benefits, while our study demonstrates that a proxy decision-maker is rarely involved.

Many clinicians in our study believed that patients with acute ischemic stroke are unable to make a well-considered treatment decision because of the acute situation, neurological deficits, and time-pressure. Although international guidelines recommend to discuss risks and benefits of tPA treatment, if a patient with disabling acute ischemic stroke cannot provide consent and a legal representative is not immediately available it is considered justified to start treatment without any form of consent [35–37]. Besides, since thrombolysis is a standard emergency treatment in acute ischemic stroke, one can even argue that thrombolysis might be started without informed consent [33, 36]. Multiple studies have proven the efficacy of tPA treatment in acute ischemic stroke and even show promising use in a selection of patients outside of the 4.5 h window [10, 38].

Besides aphasia and lowered consciousness, not directly apparent neurological deficits resulting from stroke, such as cognitive impairments, may reduce the decision-making capacity of patients with stroke [39]. These deficits may seriously hinder the informed consent process if the goal of this process is to make the patient truly understand the issue at hand at an acceptable level, and give consent for treatment based on this information. Instead, it might be desirable to shift from informed consent to *informed refusal* of tPA treatment in acute ischemic stroke [33]. Instead of actively asking for consent, informed refusal leaves room for patients to refuse treatment after being informed about its risks and benefits.

The shift from informed consent to informed refusal may have medical, ethical and legal implications. Firstly, legal concerns might drive the informed consent practice in its current form. If patients are not asked for their consent for treatment, the responsibility of treatment and its potential complications might shift towards the physician. Previous studies suggested that asking and documenting informed consent may give the clinician some legal protection [40]. However, most studies investigating the litigation of thrombolysis in acute ischemic stroke were performed in the United States, and their external validity to other countries is debatable [40, 41]. Interestingly, the majority of this litigation is related to failure to administer tPA treatment, instead of treatment complications [40]. The Dutch law states that treatments can be performed without consent in an emergency situation, if there is no time for obtaining consent because delayed treatment results in serious harm to the patient. As one might argue that this is the case in tPA treatment, informed refusal seems to be allowed from a legal perspective. Secondly, ethical issues play a role, as patient autonomy and involvement in decision making might be threatened with this practice. However, informed refusal does not fully eliminate the patient’s involvement in the decision-making process. Where appropriate, the patient can decide to refuse treatment based on the information that is provided. Additionally, for tPA treatment in acute ischemic stroke, there is no real viable alternative treatment and therefore lacks a preference-sensitive choice that can be used in shared decision making [33]. Thirdly, medical aspects such as stroke severity or decision-making capacity play a role in the informed consent process. Therefore, it may be appropriate to consider these medical aspects for each patient individually, and if there are situations where it is debatable if benefits of treatment outweigh its risks (e.g. in patients with very low stroke severity with good prognosis), provide more information and leave more room for the patient’s preferences.

A limitation of this study is that, due to its nature as a survey, socially desirable responses may have been provided in the questionnaire and clinicians may be unrealistically optimistic about their own informed consent practice. Additionally, it is difficult to capture the actual clinical practice and its nuances in closed-ended questions. These factors could have led to suboptimal reflection of daily practice. Another limitation to our study is the moderate sample size and the underrepresentation of neurologists. However, this distribution of residents and attending neurologists may be an adequate reflection of the composition of most neurology departments. Neurologists and residents received a different questionnaire to reflect their actual clinical practice and thus the questionnaires had to be analyzed separately for neurologists and residents. Although this may have reduced the likelihood of uncovering differences in practice, this gave particular understanding of the actual practice of neurologists and residents, instead of the desired or expected practice. Because laws, ethics, and medical guidelines may differ per country, we were unable to formulate a universal advice regarding the recommended practice of informed consent or informed refusal. However, we think that the discussion of these aspects may provide the necessary resources for clinicians to determine the practice that best suits their situation.

A strength of this study is that we investigated the clinicians’ informed consent practice in four high volume stroke centers both quantitatively and qualitatively. Therefore, we were able to provide more detailed insight into the meaning of the collected quantitative data. Another strength of our study was the high response rate compared to similar studies using digital questionnaires among healthcare providers [26].

## Conclusions

The current practice of obtaining informed consent for thrombolysis in acute ischemic stroke varies considerably in our study. We found that the majority of clinicians reported to use less than one minute for obtaining informed consent in acute ischemic stroke patients eligible for thrombolysis. In our opinion, it is nearly impossible to obtain valid informed consent in this situation, which causes friction between legal issues, ethical issues, and medical concerns. Therefore, we suggest a shortened information provision process, making a shift from informed consent to informed refusal, while still considering the patient’s capacity, level of neurological impairment, and possible treatment delays.

## Supplementary Information

**Additional file 1.** Study questionnaires.

## Data Availability

The datasets used and analysed during the current study are available from the corresponding author on reasonable request.

## Abbreviations

AMC: Academic Medical Center
ER: Emergency room
ICH: Intracerebral haemorrhage
IQR: Interquartile range
IVT: Intravenous thrombolysis
NA: Not applicable
QDA: Qualitative data analysis
SD: Standard deviation
SPSS: Statistical Package for the Social Sciences
tPA: Tissue-type Plasminogen Activator
UMC: University Medical Centers
VUmc: VU Medical Center
