# Supplementary material for: Practice variation in the informed consent procedure for thrombolysis in acute ischemic stroke: a survey among neurologists and neurology residents
Source: BMC Med Ethics. 2021 Aug 25;22:114. doi: 10.1186/s12910-021-00684-6 (PMC8390276; doi:10.1186/s12910-021-00684-6)
Supplement: Supplementary file 1 — Additional file 1. Study questionnaires. [file 12910_2021_684_MOESM1_ESM.docx]

**Supplementary file – Study questionnaires**

Intro:

The questions in this survey concern the administration of tPA treatment in patients with acute ischemic stroke, within 4.5 hours after stroke onset.

Thank you for your time!

**Questionnaire for neurology residents**:

Please note: all questions regard your standard practice:

1. I administered tPA in this many patients with acute ischemic stroke:
   1. 0 patients
   2. 1-25 patients
   3. >25 patients
2. I inform patients about the diagnosis ischemic stroke before starting tPA treatment:
   1. Always
   2. Often
   3. Sometimes
   4. Never
3. I inform patients about the mechanism of action of tPA treatment before starting tPA treatment:
   1. Always
   2. Often
   3. Sometimes
   4. Never
4. I inform patients about the risks of tPA treatment before starting tPA treatment:
   1. Always
   2. Often
   3. Sometimes
   4. Never
5. The risks that I discuss with the patient before starting tPA treatment are: (more than one answer possible)
   1. The chance for an intracerebral hemorrhage
   2. Other, namely:
6. I inform patients about the benefits of tPA treatment before starting tPA treatment:
   1. Always
   2. Often
   3. Sometimes
   4. Never
7. The benefits that I discuss with the patient before starting tPA treatment are: (more than one answer possible)
   1. Improved functional outcome
   2. Other, namely:
8. I provide every patient with the same information about tPA treatment:
   1. Yes, skip question 9
   2. Nee
9. Why do you provide patients with different information about tPA treatment?
10. Before starting tPA treatment, I ask the patient for explicit consent to start tPA treatment:
    1. Always
    2. Often
    3. Sometimes
    4. Never
11. If a patient is not capable of providing informed consent for tPA treatment:
    1. I start tPA treatment, if no contraindications are present
    2. I obtain proxy consent, if a proxy is present in the ER
    3. I obtain proxy consent, even if no proxy is present on the ER
    4. I refrain from tPA treatment
12. The time I spend on informing the patient and obtaining consent from the patient for tPA treatment is:
    1. 0 minutes
    2. 0-1 minutes
    3. 1-5 minutes
    4. >5 minutes
13. Obtaining informed consent causes a delay in starting tPA treatment:
    1. Always
    2. Often
    3. Sometimes
    4. Never, skip question 14
14. The delay in start of tPA treatment because of informed consent causes otherwise preventable harm in patients with acute ischemic stroke:
    1. Always
    2. Often
    3. Sometimes
    4. Never
    5. Unknown
15. A patient with an acute ischemic stroke is able to make a well considered decision about starting tPA treatment when informed about the risks and benefits of tPA treatment:
    1. Always
    2. Often
    3. Sometimes
    4. Never
16. Approximately this many of my patients have refused tPA treatment:
    1. 0 patients
    2. 1-5 patients
    3. >5 patients
17. Can you please share your considerations whether to obtain or not obtain informed consent for tPA treatment in patients with an acute ischemic stroke?
18. **(optional)** Do you have remarks or questions about this survey or the subject informed consent for tPA treatment that you would like to share?

**Questionnaire for neurologists**:

Please note: all questions regard your standard practice:

1. I supervised tPA treatment in this many patients with acute ischemic stroke:
   1. 0 patients
   2. 1-25 patients
   3. >25 patients
2. Under my supervision, the resident discusses the diagnosis ischemic stroke with the patient before starting tPA treatment:
   1. Always
   2. Often
   3. Sometimes
   4. Never
   5. Unknown
3. Under my supervision, the resident discusses the mechanism of tPA treatment with the patient before starting tPA treatment:
   1. Always
   2. Often
   3. Sometimes
   4. Never
   5. Unknown
4. Under my supervision, the resident discusses the risks of tPA treatment with the patient before starting tPA treatment:
   1. Always
   2. Often
   3. Sometimes
   4. Never
   5. Unknown
5. Under my supervision, the resident discusses the following risks of tPA treatment with the patient before starting tPA treatment:
   1. The risk of an intracerebral hemorrhage
   2. Other, namely:
   3. Unknown
6. Under my supervision, the resident discusses the benefits of tPA treatment with the patient before starting tPA treatment.
   1. Always
   2. Often
   3. Sometimes
   4. Never
   5. Unknown
7. Under my supervision, the resident discusses the following benefits of tPA treatment with the patient before starting tPA treatment:
   1. An improved functional outcome
   2. Other, namely:
   3. Unknown
8. I expect the resident to always discuss the same risks and benefits of tPA treatment with patients:
   1. Yes
   2. No
9. Why do you expect the resident to discuss different risks and benefits of tPA treatment with patients?
10. Under my supervision, the residents asks the patient for explicit consent to start tPA treatment:
    1. Always
    2. Often
    3. Sometimes
    4. Never
    5. Unknown
11. If under my supervision a patient is incapable of providing consent for tPA treatment, the resident acts as follows:
    1. Starts tPA treatment, if no contraindications are present
    2. Obtains proxy consent, if a proxy is present in the ER
    3. Obtains proxy consent, even if no proxy is present on the ER
    4. Refrains from tPA treatment
    5. Unknown
12. I believe it is necessary to spend this many minutes on informing the patient and obtaining consent before starting tPA treatment:
    1. 0 minutes
    2. 0-1 minutes
    3. 1-5 minutes
    4. >5 minutes
13. Obtaining informed consent causes a delay in starting tPA treatment:
    1. Always
    2. Often
    3. Sometimes
    4. Never
    5. Unknown
14. The delay in start of tPA treatment because of informed consent, causes otherwise preventable harm in patients with acute ischemic stroke:
    1. Always
    2. Often
    3. Sometimes
    4. Never
    5. Unknown
15. A patient with an acute ischemic stroke is able to make a well considered decision about starting tPA treatment, when informed about the risks and benefits of tPA treatment:
    1. Always
    2. Often
    3. Sometimes
    4. Never
16. Approximately this many of my patients have refused tPA treatment:
    1. 0 patients
    2. 1-5 patients
    3. >5 patients
    4. Unknown
17. Can you please share your considerations whether to obtain or not obtain informed consent for tPA treatment in patients with an acute ischemic stroke?
18. **(optional)** Do you have remarks or questions about this survey or the subject informed consent for tPA treatment that you would like to share?
